# Photon counting computed tomography of in-stent-stenosis in a phantom: Optimal virtual monoenergetic imaging in ultra high resolution

**DOI:** 10.1016/j.heliyon.2024.e27636

**Published:** 2024-03-09

**Authors:** Arwed Elias Michael, Denise Schoenbeck, Matthias Michael Woeltjen, Jan Boriesosdick, Julius Henning Niehoff, Alexey Surov, Jan Borggrefe, Bernhard Schmidt, Christoph Panknin, Tilman Hickethier, David Maintz, Alexander Christian Bunck, Roman Johannes Gertz, Jan Robert Kroeger

**Affiliations:** a Department of Radiology, Neuroradiology and Nuclear Medicine, Johannes Wesling University Hospital, Ruhr University Bochum, Bochum, Germany; b Siemens Healthineers, Erlangen, Germany; c Institute for Diagnostic and Interventional Radiology, University of Cologne, Faculty of Medicine and University Hospital Cologne, Cologne, Germany

**Keywords:** Computed tomography, Photon counting computed tomography, Photon counting detector, In-stent stenosis, Stent imaging, Virtual monoenergetic image

## Abstract

**Rationale and objectives:**

Coronary computed tomography angiography (CCTA) is becoming increasingly important for the diagnostic workup of coronary artery disease, nevertheless, imaging of in-stent stenosis remains challenging. For the first time, spectral imaging in Ultra High Resolution (UHR) is now possible in clinically available photon counting CT. The aim of this work is to determine the optimal virtual monoenergetic image (VMI) for imaging in-stent stenoses in cardiac stents.

**Materials and methods:**

6 stents with inserted hypodense stenoses were scanned in an established phantom in UHR mode. Images were reconstructed with 3 different kernels for spectral data (Qr56, Qr64, Qr72) with varying levels of sharpness. Based on region of interest (ROI) measurements image quality parameters including contrast-to-noise ratio (CNR) were analyzed for all available VMI (40 keV–190 keV). Finally, based on quantitative results and VMI used in clinical routine, a set of VMI was included in a qualitative reading.

**Results:**

CNR showed significant variations across different keV levels (p < 0.001). Due to reduced noise there was a focal maximum in the VMI around 65 keV. The peak values were observed for kernel Qr56 at 116 keV with 19.47 ± 8.67, for kernel Qr64 at 114 keV with 13.56 ± 6.58, and for kernel Qr72 at 106 keV with 12.19 ± 3.25. However, in the qualitative evaluation the VMI with lower keV (55 keV) performed best.

**Conclusions:**

Based on these experimental results, a photon counting CCTA in UHR with stents should be reconstructed with the Qr72 kernel for the assessment of in-stent stenoses, and a VMI 55 keV should be computed for the evaluation.

## 1 Introduction

Coronary artery disease (CAD) has a high prevalence and mortality [1]. Coronary computed tomography angiography (CCTA) is increasingly important for the noninvasive diagnosis of CAD [2]. Initially CCTA was primarily used for the examination of patients with suspected but not yet confirmed CAD. In recent years the range of applications for CCTA has expanded considerably. For example, the assessment of the hemodynamic relevance of stenoses using CT-derived fractional flow reserve has already been validated and introduced into clinical practice. The use of CCTA for planning percutaneous coronary interventions is also promising [3]. As a result, more and more patients with known CAD and often with coronary stents are examined using CCTA. The examination of stents and particularly the assessment of in-stent stenoses by CCTA remains a challenge that could not be solved satisfactorily even with the latest dual-source CT scanners [4]. False positive results or inconclusive findings regarding in-stent stenosis still occur in about one third of patients [4]. Technical advances in photon counting computed tomography (PCCT), which has recently been introduced into clinical practice, should further improve examination quality of CCTA by providing data with higher resolution, less noise, and inherent spectral information [5,6]. The first studies already indicate that image quality and diagnostic confidence [7] can be improved along with better plaque characterization [8] in CCTA. Recent findings also suggest a considerable potential for improvement in post-processing, with optimized reconstruction kernels offering further advancements in the imaging of cardiac stents [9].

Two different resolution modes are currently available for PCCT: the normal resolution mode (NRM) with a total collimation of 144 × 0.4 mm and a focal spot size 0.8 × 1.1 mm, and the ultra high resolution mode (UHR) with a total collimation of 120 × 0.2 mm and a focal spot size of 0.6 × 0.7 mm. Until now, spectral data acquisition was only possible for the NRM [9]. With the latest hardware and software updates, spectral imaging is now also available for the UHR with the caveat of a reduced collimation of 96 × 0.2 mm. The considerably higher spatial resolution in UHR also results in significantly improved imaging of the stent lumen in stents [9], therefore CCTA should be performed in UHR in patients with cardiac stents. Based on the spectral information in PCCT, virtual monoenergetic images, superior to the polyenergetic images in CCTA, can be calculated [10]. For this reason, determining the optimal monoenergetic image in UHR is of utmost importance for the most accurate evaluation of stents, especially in-stent stenoses.

This experimental work was undertaken to systematically evaluate different monoenergetic images using UHR photon counting scans in an established phantom in terms of the ability to assess in-stent stenoses.

## 2 Material and methods

### 2.1 Phantom of in-stent stenosis

Institutional review board approval was obtained. An established model [9,11] was used to evaluate in-stent stenoses (for a diagram please refer to the supplementary material). In this model, the coronary vessel was represented by a plastic tube with a diameter of approximately 3 mm. The wall thickness of the plastic tube was about 0.3 mm with a density of 35 HU, which is in the soft tissue range comparable to the human vessel wall. Six different stents of different materials and strut thicknesses (see Table 1) were selected to match the variety of stents expected in the current patient population undergoing CCTA. The stents were placed in the center of the plastic tubes. The artificial hypodense stenoses were made of a wax-based material mixed with a lipophilic contrast agent (Lipiodol Ultra-Fluid; Guerbet GmbH, Sulzbach, Germany) titrated to measure 45 HU at 120 kVp [11]. A small portion of the wax-based material was positioned in the lumen of the stent. The material was then pressed and hereby fixed to the stent strut using a 1.5-mm balloon catheter (Armada 14; Abbott GmbH, Wiesbaden, Germany) over a microwire (V-14 Control Wire; Boston Scientific GmbH, Ratingen, Germany). The aim was to create stenoses between 50 % and 75 %, though, due to the manufacturing technique, a variance of the resulting artificial stenoses was unavoidable. Therefore, the actual extent of the artificially produced in-stent stenoses had to be angiographically determined (according to the clinical reference standard) and appropriate diameter stenoses between 50 % and 70% were confirmed (see Table 1).

**Table 1:** Stent Characteristics.

name	manufacturer	material	diameter [mm]	length [mm]	strut thickness [mm]	stenosis [%]
Chrono	Sorin Biomedical	CoCr	3	20	0.08	68.94
Coroflex Please	Braun	StSt 316 L	3	19	0.12	57.81
Endeavor	Medtronic	CoCr	3	30	0.091	69.10
Promus Element Plus	Boston Scientific	PlCr	3	19	0.081	67.62
Radius	Boston Scientific	Nitinol	3	20	0.085	53.80
Tantal Coronary	Abbott/Guidant	Tantalum	3	19	0.058	66.10

StSt: Stainless Steel; CoCr: Cobalt Chrome; PlCr: Platinum Chromium.

### 2.2 Image acquisition and reconstruction

The plastic tube was filled with a solution of saline (0.9 %) and contrast agent (300 mg iohexol/ml, Accupaque 300, GE Healthcare), adjusted in order to measure 400 HU in a polyenergetic reconstruction of a scan with 120 kVp in the PCCT. This density corresponds to the average contrast of a CCTA according to our clinical experience. The tube was then sealed airtight on both sides and placed in a plastic container measuring (length) 36 cm x (width) 24 cm x (height) 14 cm, likewise filled with saline (0.9 %). This container was placed at an angle of 45°to the z-axis [9] in the gantry of the scanner such that the plastic tube with the stent came into position slightly below the isocenter.

The phantom was examined in a dual source photon counting CT (NAEOTOM Alpha, software version Syngo CT VB10, Customer Use Test version, Siemens Healthineers, Erlangen, Germany) with a clinical CT protocol. Sequential scans were obtained for each stent in UHR. The tube voltage was set at 120 kVp and the effective tube current at 50 mAs, matching the average of CCTA examinations performed at our hospital. The total collimation was 96 × 0.2 mm, the focal spot size 0.6 × 0.7 mm. The Quantumplus mode, for the first time achievable for the UHR, allowed for the acquisition of spectral data and generation of virtual monoenergetic images.

For each in-stent stenosis 3 primary reconstructions were prepared using the kernels Qr56, Qr64 and Qr72, respectively. These kernels were designed for spectral data and differ merely regarding their sharpness. Slice thickness was set to the minimum of 0.2 mm, increment to 0.2 mm. The matrix was set to 1024 x 1024, and the Field of View (FOV) was narrowed to about 100 mm × 100 mm, so that the stent lying slightly outside the isocenter was well imaged with surrounding fluid on each side. The third level of iterative reconstruction (QIR, Quantum Iterative Reconstruction, four levels of iteration) was applied, as currently performed in our routine clinical practice. Based on each thin-layered primary reconstruction, strictly axial and longitudinal multiplanar reconstructions were generated for further analysis deploying the manufacturer specific spectral workstation (Syngo.Via, VB80 (Customer Use Test version), Siemens Healthineers, Erlangen, Germany) with the minimal slice thickness of 0.1 mm and an increment of 0.1 mm. For these multiplanar reconstructions, all possible VMI could be calculated (40 keV–190 keV, i. e. 151 different keV levels).

### 2.3 Quantitative image analysis

Two experienced readers (5 and 7 years of experience in cardiac CT) performed ROI-based measurements according to prior studies [11]. In the longitudinal reconstruction, the first ROI [1] was set in the contrast-filled tube outside the stent, the second ROI [2] within the visible stent lumen prior to the stenosis, ROI [3] within the plaque and ROI [4] within the surrounding saline. Every ROI's mean density and its standard deviation were documented for every available VMI ranging from 40 keV to 190 keV. The signal of a ROI was defined as its mean density (CT value) and the noise as its standard deviation in Hounsfield units. The standard deviation of the density of the surrounding saline was considered as the image noise. The contrast to noise ratio (CNR) of stent lumen and plaque was defined as the difference of the mean attenuation between the contrasted stent lumen and the plaque divided by the image noise.

### 2.4 Qualitative image analysis

The qualitative analysis of the images was performed by six readers: two senior radiologists with 20 and 9 years of experience, four residents with 5, 4, 3 and 2 years of experience in cardiac CT, respectively.

**Fig. 1:**
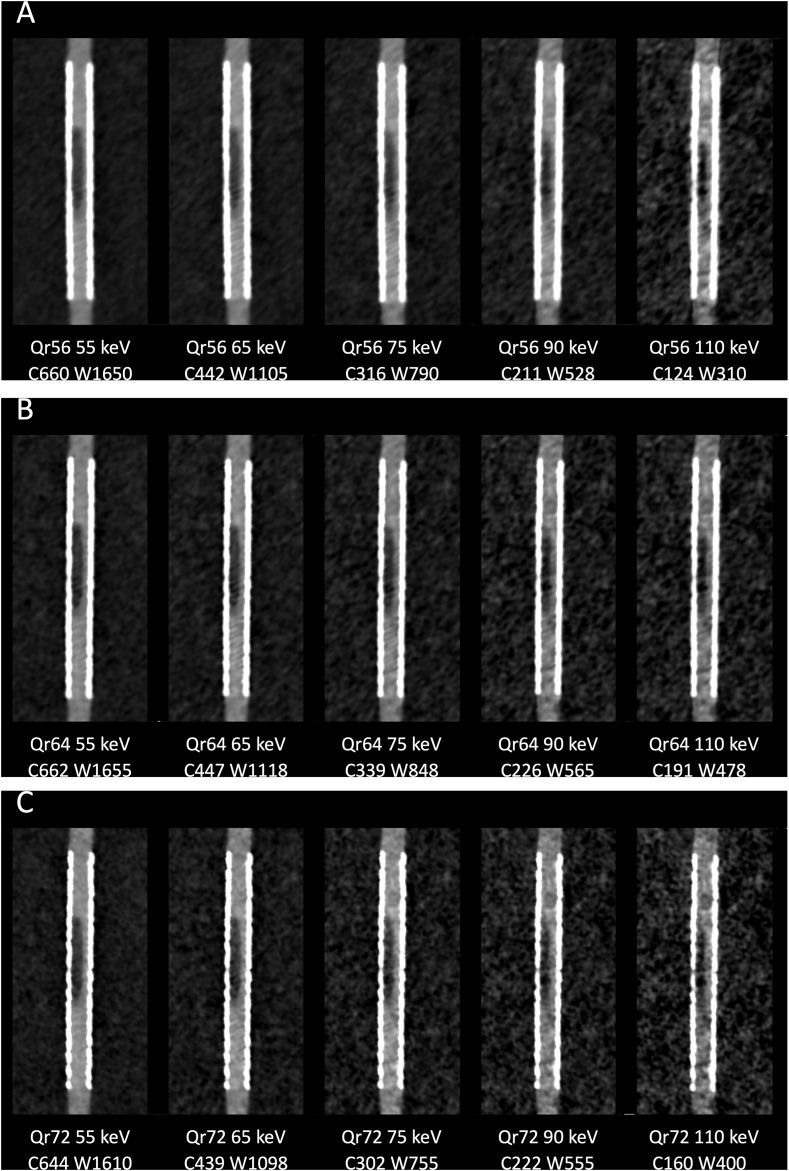
**Longitudinal Reconstructions with Qr Kernels.** The figure shows reconstructions of the Endeavor stent using the kernel Qr56 (A), Qr64 (B) and Qr72 (C), respectively. For each kernel, five VMIs are calculated, namely 55 keV, 65, keV, 75 keV, 90 keV and the keV with the maximum CNR in the quantitative analysis. Window settings are optimized for each VMI. VMI: virtual monoenergetic images; keV kiloelectron volt; C: window center; W: window width.

For each reconstruction of a stent with kernel Qr56, Qr64, or Qr72, five VMIs were calculated: 55 keV as the VMI in current clinical routine, 65 keV, 75 keV, and 90 keV as intermediate levels, and 110 keV, close to the maxima of the CNR in quantitative analysis (see Fig. 1). These VMIs were displayed in a longitudinal orientation optimal for the assessment of the in-stent stenosis. The window was individually optimized for each VMI using a ROI in the contrasted tube outside the stent: the mean density of the ROI was chosen as the center, and 2.5 times the mean density as the width [12]. This window for each VMI was preset and fixed for all readers. The readers rated the assessability of the stenosis on a 5-point Likert scale (1: “stenosis not assessable” to 5: “stenosis perfectly assessable”). The readers were blinded regarding the kernel used as well as the energy level of the VMI, the rating was carried out independently of each other.

### 2.5 Statistical analysis

Data curation, data processing and statistical analyses were performed using the statistical software R (Version 4.1.0; 13) and RStudio (Version 2022.07.1 + 554, Posit PBC, Boston, USA). A p-value of <0.05 was considered statistically significant.

The Shapiro–Wilk test was applied to test for normal distribution, the Levene test to check for homoscedasticity. When comparing multiple means a one-way ANOVA or the Friedman test as the corresponding nonparametric method was used. Post hoc analyses were carried out with T-Test and Wilcoxon test. P values were corrected according to the Bonferroni method. For the determination of the interreader reliability of the ordinal Likert ratings the intraclass correlation coefficient (ICC; ICC(C,1), two-way mixed, single measures, consistency) was applied. If not stated otherwise, all data are presented as mean ± standard deviation.

## 3 Results

### 3.1 Quantitative analysis: ROI based measurements

The signal in the contrasted tube outside the stent was highest at the low keV levels and decreased with increasing keV, resulting in significant signal differences here (corrected p < 0.001). Exemplarily, the signal in the reconstruction with the kernel Qr56 at 40 keV was 1148.82 ± 39.7 HU, and then at 190 keV the signal had dropped to 50.64 ± 20.15 HU. There were no differences between the reconstruction kernels Qr56, Qr64 and Qr72 concerning the signal (for additional graphs of the signal of the contrasted lumen and the plaque please refer to the supplemental material). The signal inside the stent was significantly higher than outside the stent, especially at the low keV levels. Again, as an example for the kernel Qr56, it amounted to 1329.97 ± 90.34 HU at 40 keV and 176.39 ± 175.24 HU at 190 keV. The signal difference between the contrasted lumen of the stent and the hypodense plaque behaved congruently in the different reconstruction kernels. It was highest at low keV levels and then decreased monotonically, as exemplified for kernel Qr72 at 40 keV with 967.86 ± 156.15 HU, at 190 keV only 91.77 ± 70.89 HU.

**Fig. 2:**
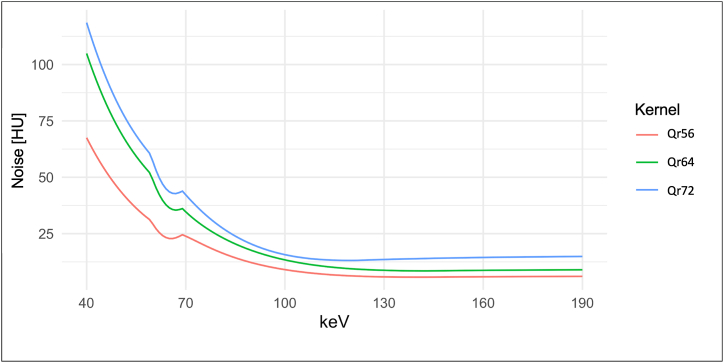
**Image Noise in VMI Using the Reconstruction Kernels Qr56, Qr64 and Qr72.** The graph shows the averaged general image noise, defined as the standard deviation in HU of the ROI in the saline surrounding the vessel phantom. The noise increases with kernel sharpness and lower keV level. VMI: Virtual Monoenergetic Reconstruction; HU: Hounsfield Units; keV: kiloelectron volt.

There were also significant differences in noise (Fig. 2) between the keV levels within a kernel (corrected p < 0.001). For example, the noise in the reconstruction with kernel Qr56 at 40 keV was 67.53 ± 10.36 HU, the lowest noise was reached at 141 keV (5.72 ± 1.83 HU). For the range between 60 and 70 keV a focal noise reduction was found compared to the adjacent keV levels. With respect to noise, differences existed for the three different reconstruction kernels. As expected, the noise increased with increasing sharpness of the kernels (Qr72 > Qr64 > Qr56), especially for the low keV levels significant differences were found (corrected p < 0.001).

**Fig. 3:**
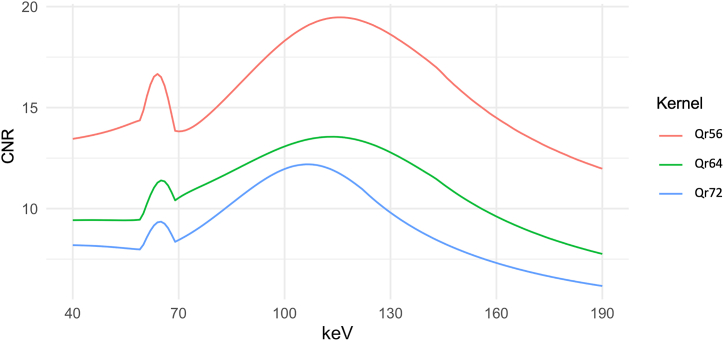
**Contrast to Noise Ratio of In-Stent Stenoses as a Function of keV Level.** The graph shows the averaged CNR in the reconstructions with different Qr kernels in all available VMI (keV level from 40 keV to 190 keV). CNR: contrast to noise ratio; keV: kiloelectron volt.

Also, the CNR differed significantly within different keV levels (p < 0.001). The CNR was low at the lower keV spectrum. It then initially increased with higher keV and had a focal maximum between 60 and 70 keV (see also Fig. 3). Depending on the reconstruction kernel, the absolute maximum of the CNR was found at a different keV level. For kernel Qr56 the maximum was at 116 keV with 19.47 ± 8.67, for kernel Qr64 at 114 keV with 13.56 ± 6.58, and finally for kernel Qr72 at 106 keV with 12.19 ± 3.25. In post hoc testing there was no significant difference between the high CNR at 65 keV and the maximal CNR at > 100 keV in each kernel.

### 3.2 Qualitative analysis: reading

**Fig. 4:**
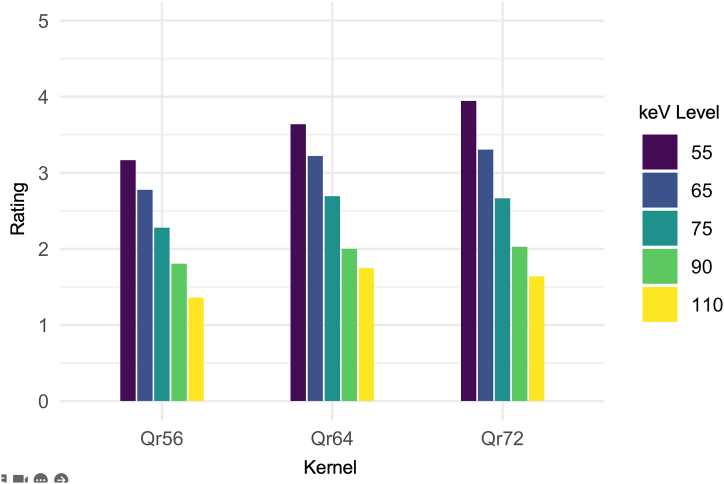
**Results of the Qualitative Analysis.** Six readers rated the assessability of the stenosis on a 5-point Likert scale (1: “stenosis not assessable” to 5: “stenosis perfectly assessable”). The graph shows the averaged rating for every selected VMI for the reconstructions with the kernels Qr56, Qr64 and Qr72 respectively. The maximum keV level is 117 keV for Qr56, 95 keV for Qr64 and 107 keV for Qr72. VMI: virtual monoenergetic image; keV: kiloelectron volt.

The readers' rating of the assessability of the stenoses in the different reconstructions and VMI achieved excellent overall consistency, the ICC being 0.75 [95% confidence interval 0.68; 0.81]. Significant differences were found between the ratings of the different reconstructions and VMI (corrected p < 0.001). For all kernels, it held equally true that the low keV VMI (55 keV) performed best (see Fig. 4). The best overall rating was given to the VMI 55 keV in the reconstruction with the kernel Qr72 (3.94 ± 1.15). In post-hoc testing, this reconstruction was significantly superior (corrected p = 0.038) to the reconstruction with the second best rating (Qr64 55 keV).

## 4 Discussion

The objective of this experimental study was to systematically determine the best monoenergetic image for the purpose of evaluating in-stent stenosis using PCCT and UHR. In this first phantom study combining UHR and spectral imaging for CCTA, we presented different findings. First quantitatively, the maximum CNR was found at higher keV levels above >100 keV. Further, a very high CNR was found at 65 keV in all kernels with no significant difference to the absolute maximum. In the qualitative analysis the VMI with lower keV of 55 keV performed best; overall the reconstruction using kernel Qr72 and the VMI at 55 keV was rated best for the assessment of the in-stent stenosis.

**Fig. 5:**
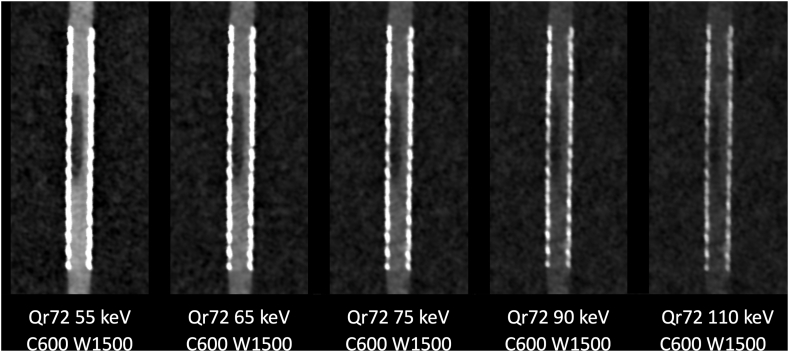
**Loss of Iodine Contrast in VMI from Low keV to High keV.** The fixed window with center 600 and width 1500 shows the evolution of contrast and noise over different VMI. With increasing keV, the contrast between the lumen of the stent and the stenosis and the surrounding area, which is based on iodine, is increasingly lost. The noise also decreases substantially. In the qualitative analysis however, a high contrast seemed more important than low noise. VMI: virtual monoenergetic image; keV: kiloelectron volt; C: window center; W: window width.

The slight discrepancy between the qualitative and quantitative analysis seems noticeable. While the CNR quantitatively maximizes both at VMI with keV around 65 and shortly above 100, the reading is fairly unequivocal and favors the VMI with lower keV. This might be attributed to the fact that human readers prioritize higher contrast over lower noise when assessing an in-stent stenosis (see also Fig. 5). Moreover, the use of a wider image window allowed by the great signal in low-keV VMI, decreases the visual conspicuity of the noise. The result of the qualitative analysis of this study is comparable to the data from a study with a preclinical PCCT [11]. Irrespective of the differences between the various CT systems, here the VMI with low keV is also preferred for the assessment of in-stent stenoses. Alike the authors assume that the increased contrast plays the decisive role here.

The value of VMI for CCTA has been proven for various dual-energy CT and PCCT scanners [[13], [14], [15]]. Few studies have looked at the impact of VMI and optimal keV level in the presence of cardiac stents [16,17]. However, these studies were carried out with dual energy CT systems using energy integrating detectors, incapable of generating comparable UHR imaging. The PCCT, which has recently become available for clinical use offers unique capabilities for UHR imaging that are particularly suitable for visualizing small structures like coronary stents or plaques [8,9]. Until now in this PCCT system it has not been able to combine UHR and spectral imaging for cardiac applications, so the operator had always to choose between UHR and spectral imaging. Here we present the first results combining UHR and spectral imaging for CCTA. But our study has several limitations. First of all, its design is very experimental and the transfer of these results to patient imaging is uncertain. Second, the experimental model was limited to in-stent stenoses, stent occlusions or stent fractures were not included. Moreover, we selected reconstruction kernels based on clinical experience, other kernels might yield different results. Finally, we did not evaluate the effect of different levels of iterative reconstruction.

In conclusion, based on the experimental results of our study, a photon counting CCTA in UHR with stents should be reconstructed with the Qr72 kernel. Additionally, a VMI 55 keV should be computed for the assessment of in-stent stenosis.

## CRediT authorship contribution statement

**Arwed Elias Michael:** Writing – review & editing, Writing – original draft, Visualization, Investigation, Formal analysis, Data curation, Conceptualization. **Denise Schoenbeck:** Investigation. **Matthias Michael Woeltjen:** Investigation. **Jan Boriesosdick:** Investigation. **Julius Henning Niehoff:** Supervision, Investigation. **Alexey Surov:** Writing – review & editing, Investigation. **Jan Borggrefe:** Supervision, Software, Investigation. **Bernhard Schmidt:** Software, Resources. **Christoph Panknin:** Software, Resources. **Tilman Hickethier:** Methodology, Investigation. **David Maintz:** Methodology, Investigation. **Alexander Christian Bunck:** Methodology, Investigation. **Roman Johannes Gertz:** Methodology, Investigation. **Jan Robert Kroeger:** Writing – review & editing, Writing – original draft, Visualization, Supervision, Methodology, Investigation, Formal analysis.

## Declaration of competing interest

J. R. Kroeger received honoraria for scientific lectures from GE Healthcare and honoraria for clinical advisory board membership from Siemens Healthineers. J. Borggrefe received honoraria for scientific lectures from Siemens Healthineers and Philips Healthcare. B. Schmidt and Ch. Panknin are empoyees of Siemens Healthineers. R. J. Gertz is supported by the Cologne Clinician Scientist Program (CCSP)/Faculty of Medicine/10.13039/501100008001University of Cologne, funded by the 10.13039/501100001659Deutsche Forschungsgemeinschaft (10.13039/501100001659DFG, German Research Foundation) (Project No. 413543196), received research support from 10.13039/100004320Philips Healthcare and is member on speaker's bureau of 10.13039/100004320Philips Healthcare.
