# Supplementary material for: Photon counting computed tomography of in-stent-stenosis in a phantom: Optimal virtual monoenergetic imaging in ultra high resolution
Source: Heliyon. 2024 Mar 9;10(6):e27636. doi: 10.1016/j.heliyon.2024.e27636 (PMC10950599; doi:10.1016/j.heliyon.2024.e27636)
Supplement: Multimedia component 1 [file mmc1.docx]

Supplementary Material

Supplementary table for the original research article with the title

## Photon Counting Computed Tomography of In-Stent-Stenosis in a Phantom: Optimal Virtual Monoenergetic Imaging in Ultra High Resolution

# Methods

**Schematic Representation of the Phantom**


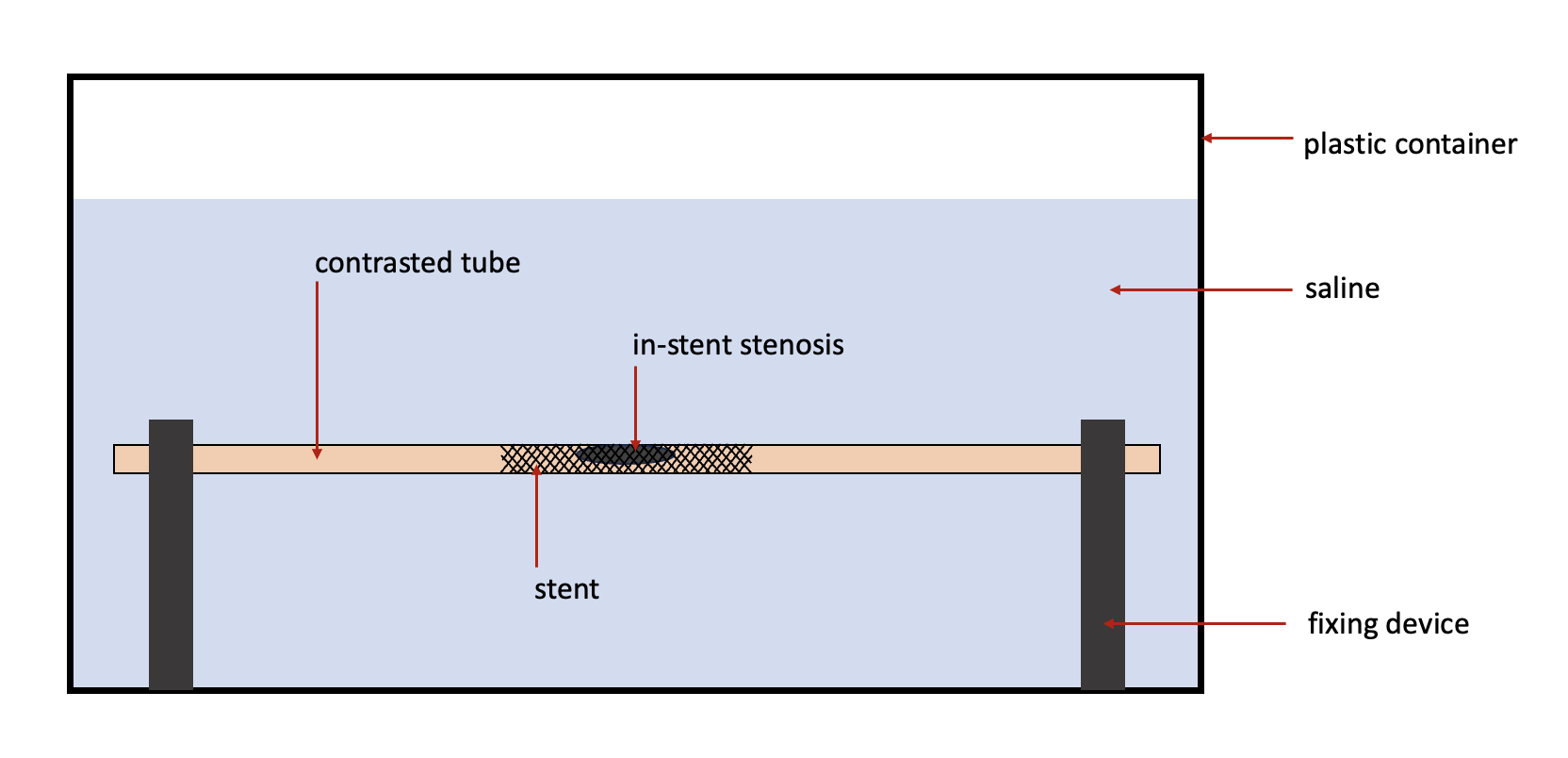


**Diagram of the Phantom:** In this model the coronary vessel was represented by a plastic tube with a diameter of approximately 3 mm. The wall thickness of the plastic tube was about 0.3 mm with a density of 35 HU, which is in the soft tissue range comparable to the human vessel wall. Different stents of different materials and strut thicknesses were placed in the center of the plastic tubes. The artificial hypodense stenoses were made of a wax-based material mixed with a lipophilic contrast agent (Lipiodol Ultra-Fluid; Guerbet GmbH, Sulzbach, Germany) titrated to measure 45 HU at 120 kVp (11). A small portion of the wax-based material was angiographically positioned in the lumen of the stent. The material was then pressed and thus fixed to the stent strut with a 1.5-mm balloon catheter (Armada 14; Abbott GmbH, Wiesbaden, Germany) over a microwire (V-14 Control Wire; Boston Scientific GmbH, Ratingen, Germany). The tube was then sealed airtight on both sides and placed in a plastic container with the help of a fixing device. The container measured (length) 36 cm × (width) 24 cm × (height) 14 cm and was filled with saline (0.9%). This container was finally placed in 45 ° angled to the z-axis (9) in the gantry of the scanner so that the plastic tube with the stent came into position slightly below the isocenter.

# Results

**Figures: Signal of Contrasted Lumen and Plaques in Different Kernels.**

| **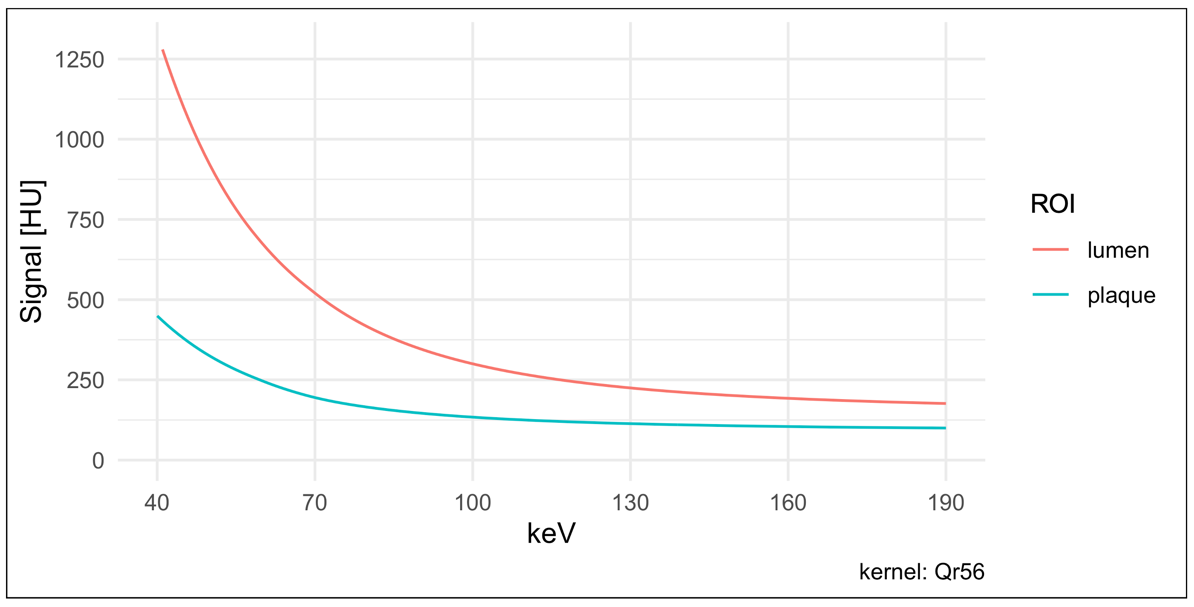** |
| --- |
| **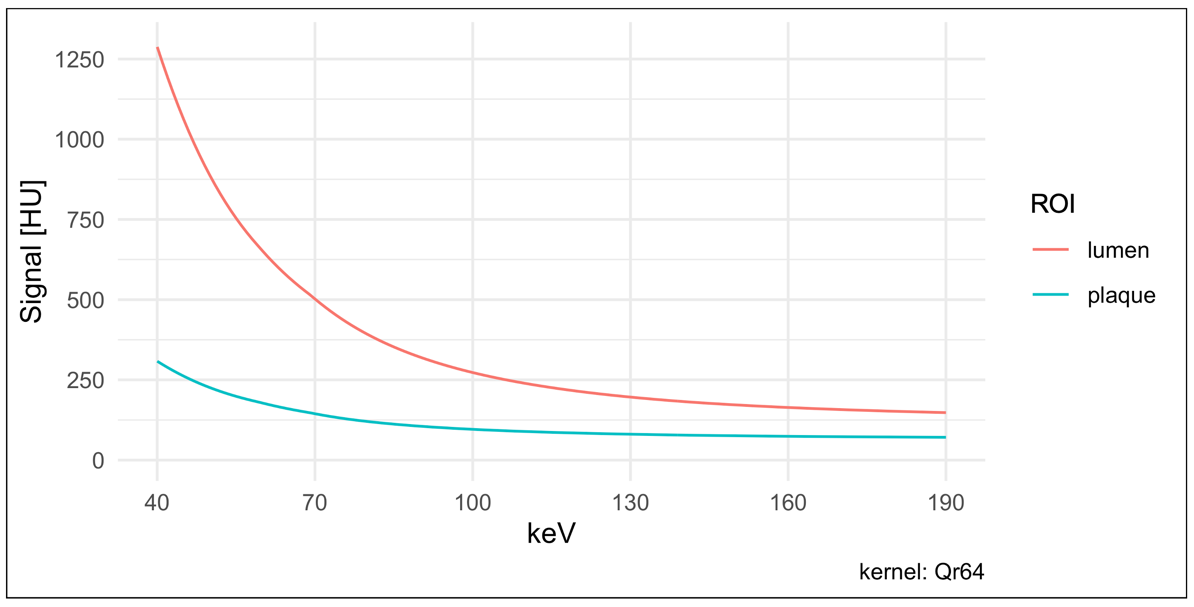** |
| **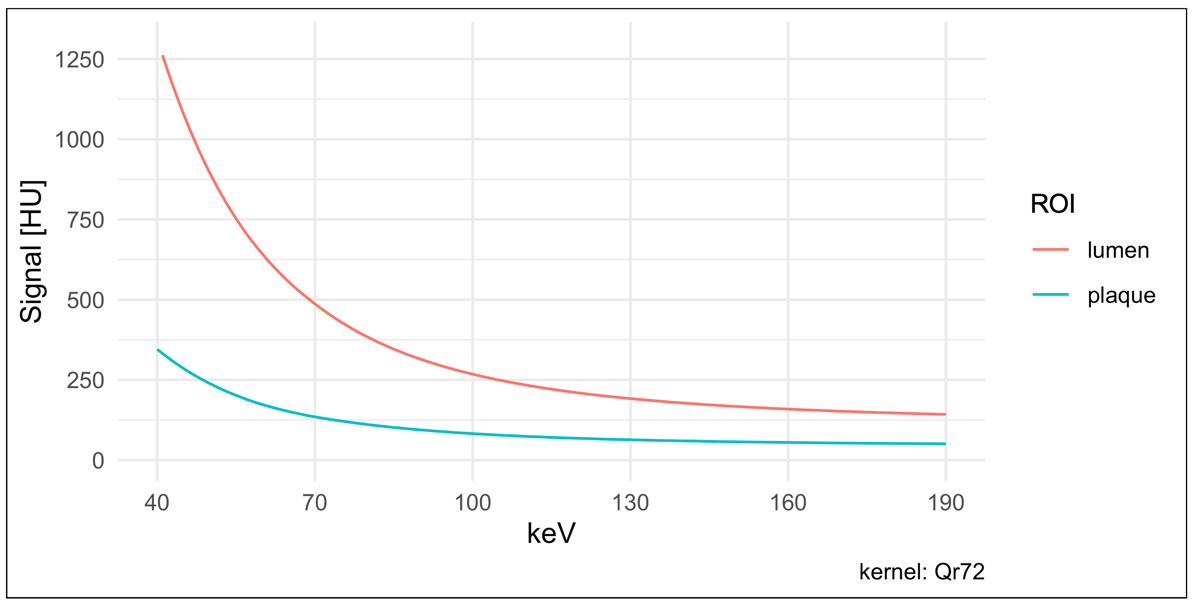** |

The graphs above show the development of the signal of the contrasted stent lumen and the hypodense plaque as a function of the keV level of the virtual monoenergetic reconstructions. For the three kernels Qr56, Qr64 and Qr72 there is an almost congruent curve.

**Table with Intraclass Correlation Coefficients**

|  | res_5 | con_20 | res_2 | res_3 | con_9 | res_4 |
| --- | --- | --- | --- | --- | --- | --- |
| res_5 |  | 0.82 | 0.76 | 0.73 | 0.76 | 0.78 |
| con_20 | 0.82 |  | 0.69 | 0.66 | 0.73 | 0.71 |
| res_2 | 0.76 | 0.69 |  | 0.82 | 0.73 | 0.76 |
| res_3 | 0.73 | 0.66 | 0.82 |  | 0.75 | 0.71 |
| con_9 | 0.76 | 0.73 | 0.73 | 0.75 |  | 0.79 |
| res_4 | 0.78 | 0.71 | 0.76 | 0.71 | 0.79 |  |
| **Supplementary Table:** intraclass correlation coefficients (ICC; ICC(C,1), two-way mixed, single measures, consistency) for all readers. res_5: resident with 5 years of experience; con_20: consultant with 20 years of experience; res_2: resident with 2 years of experience; res_3: residence with 3 years of experience; con_9: consultant with 9 years of experience; res_4: resident with 4 years of experience | | | | | | |
